# Quantum leap in medical mentorship: exploring ChatGPT’s transition from textbooks to terabytes

**DOI:** 10.3389/fmed.2025.1517981

**Published:** 2025-04-28

**Authors:** Santosh Chokkakula, Siomui Chong, Bing Yang, Hong Jiang, Juan Yu, Ruiqin Han, Idress Hamad Attitalla, Chengliang Yin, Shuyao Zhang

**Affiliations:** ^1^Department of Microbiology, Chungbuk National University College of Medicine and Medical Research Institute, Cheongju, Chungbuk, Republic of Korea; ^2^Department of Dermatology, The University of Hong Kong-Shenzhen Hospital, Shenzhen, China; ^3^Department of Dermatology, The First Affiliated Hospital of Jinan University andJinan University Institute of Dermatology, Guangzhou, Guangdong, China; ^4^Institute of Collaborative Innovation, University of Macau, Macao SAR, China; ^5^Department of Cell Biology, College of Basic Medical Sciences, Tianjin Medical University, Tianjin, China; ^6^Department of Public Health, International School, Krirk University, Bangkok, Thailand; ^7^Statistical Office, Department of Operations, Zhuhai People's Hospital, Zhuhai Clinical Medical College of Jinan University, Zhuhai, China; ^8^Department of Radiology, The First Affiliated Hospital of Shenzhen University, Health Science Center, Shenzhen Second People’s Hospital, Shenzhen, China; ^9^State Key Laboratory of Common Mechanism Research for Major Diseases, Institute of Basic Medical Sciences, Chinese Academy of Medical Sciences and Peking Union Medical College, Beijing, China; ^10^Department of Microbiology, Faculty of Science, Omar Al-Mukhtar University, AL-Bayda, Libya; ^11^Medical Innovation Research Department, Chinese PLA General Hospital, Beijing, China; ^12^Department of Pharmacy, Guangzhou Red Cross Hospital of Jinan University, Guangzhou, China

**Keywords:** artificial intelligence, ChatGPT, evidence-based medicine, healthcare technology, personalized learning, clinical problem-solving, digital literacy and privacy

## Abstract

ChatGPT, an advanced AI language model, presents a transformative opportunity in several fields including the medical education. This article examines the integration of ChatGPT into healthcare learning environments, exploring its potential to revolutionize knowledge acquisition, personalize education, support curriculum development, and enhance clinical reasoning. The AI’s ability to swiftly access and synthesize medical information across various specialties offers significant value to students and professionals alike. It provides rapid answers to queries on medical theories, treatment guidelines, and diagnostic methods, potentially accelerating the learning curve. The paper emphasizes the necessity of verifying ChatGPT’s outputs against authoritative medical sources. A key advantage highlighted is the AI’s capacity to tailor learning experiences by assessing individual needs, accommodating diverse learning styles, and offering personalized feedback. The article also considers ChatGPT’s role in shaping curricula and assessment techniques, suggesting that educators may need to adapt their methods to incorporate AI-driven learning tools. Additionally, it explores how ChatGPT could bolster clinical problem-solving through AI-powered simulations, fostering critical thinking and diagnostic acumen among students. While recognizing ChatGPT’s transformative potential in medical education, the article stresses the importance of thoughtful implementation, continuous validation, and the establishment of protocols to ensure its responsible and effective application in healthcare education settings.

## 1 Introduction

The swift advancements in artificial intelligence (AI) and natural language processing (NLP) have resulted in the development of increasingly complex language models (1, 2). Generative AI refers to a category of models that can create new content by analyzing and synthesizing patterns from existing data. These models are versatile, capable of producing outputs in various formats, such as text, images, and music (3, 4). Among these innovations, ChatGPT, a product of OpenAI, has emerged as a significant tool with applications across a wide range of fields (5–7). To fully understand its impact on scientific inquiry, it is essential to explore the model’s origins and developmental journey (8–14). It is important to note that ChatGPT operates differently from Generative Adversarial Networks (GANs). Instead, it is based on the Generative Pre-Trained Transformer (GPT) architecture (15–18). While GANs are primarily used for generating images, GPT models are specifically designed for natural language tasks, including text generation and comprehension (19–21).

The operation of the ChatGPT in medical productivity is significantly impacted by its training data and model building. The evolution of ChatGPT involved well-informed fine-tuning of large language models (LLMs) like GPT-3.5 and GPT-4. These models were trained on massive datasets, with GPT-3 using 45 terabytes of text data from various sources including Common Crawl, WebText2, Books1, Books2, and Wikipedia (22). This vast and multifarious dataset permit the models to accomplish state-of-the-art execution in a wide range of natural language processing tasks, including those applicable to medicine. The model building has also grownup progressively bigger, with GPT-3 featuring 175 billion parameters, over 100 times larger than its predecessor GPT-2. This expand in model size has led to developed few-shot and zero-shot learning abilities, permitting ChatGPT to execute well on job it wasn’t generally trained for, including medical work (23). The refining procedure, which participate strengthening learning from human feedback (RLHF) and additional autonomous adversarial training, further increased the power of ChatGPT to supply suitable and contextually applicable consequences in medical language (24). The GPT framework allows ChatGPT to produce text that closely resembles human writing by utilizing extensive training data and advanced machine learning techniques. Beyond simple text generation, ChatGPT can tackle complex language tasks, such as answering questions, providing detailed explanations, and assisting in creative writing. This adaptability has led to its use in various scientific fields, potentially transforming the approaches researchers take toward data analysis, literature reviews, and hypothesis development. However, it is crucial to critically assess the limitations and potential biases that AI models like ChatGPT may introduce. As research in this area continues to evolve, the scientific community must carefully navigate the opportunities and challenges presented by these language models, ensuring their ethical and effective application in scientific research.

**Figure 1:**
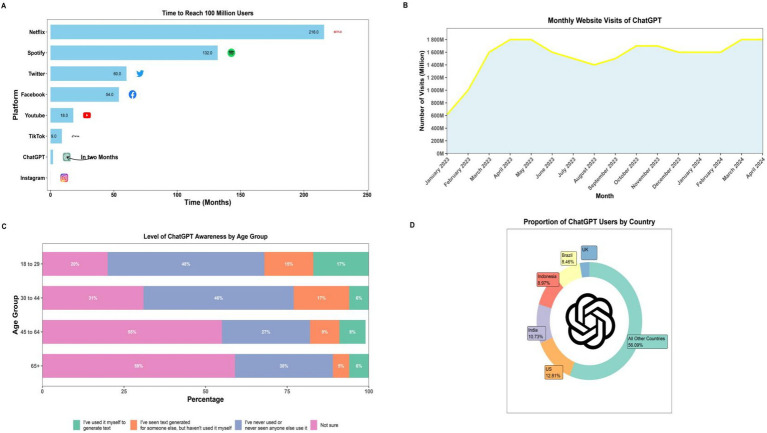
Statistical trends in ChatGPT’s online engagement. **(A)** Time to reach 100 million users of ChatGPT compared to other online platforms. **(B)** Monty website visits of ChatGPT during January 2023 to April 2024. **(C)** Levels of ChatGPT awareness among different age groups. **(D)** Proportion of ChatGPT users by country.

The emergence of advanced language models has dramatically changed the field of artificial intelligence, with OpenAI’s GPT series leading this transformation. The introduction of ChatGPT, which reached over 1 million subscribers within just 5 days of its launch, exemplifies the rapid impact of these technologies (Figure 1). This evolution began with GPT-3.5, released in late 2022, and continued with the more advanced GPT-4 in early 2023. These models have demonstrated exceptional capabilities in addressing complex inquiries across various disciplines, including medicine, which has attracted significant attention from experts in the field (7, 25). ChatGPT, Bard, and Claude represent a new generation of advanced conversational AI systems, offering users an interactive platform for querying a vast knowledge base. Since its public release, ChatGPT has captured significant media attention and rapidly accumulated a substantial user base (26). These large language models (LLMs) are trained on an extensive corpus of diverse internet-based text sources, encompassing articles, forum discussions, and websites, with additional fine-tuning on curated datasets including books and scholarly articles (27). The performance of these AI systems in specialized domains has been noteworthy. For instance, ChatGPT achieved a score of 60.2% on a set of multiple-choice questions derived from the US Medical License Exams (26). In comparison, Google’s Med-PaLM 2 demonstrated even more impressive results, scoring up to 86.5% on similar tests. The advent of these sophisticated AI chatbots may herald a transformative era in knowledge dissemination, potentially rivaling the impact of Gutenberg’s printing press (26).

A pivotal aspect of ChatGPT’s development is its training data source. Unlike specialized systems that depend on professional medical databases, ChatGPT primarily utilizes publicly available information. This methodology presents both benefits and challenges. On the one hand, it allows for a comprehensive knowledge base that spans multiple areas. On the other hand, it raises concerns regarding the model’s ability to produce responses that adhere to the principles of evidence-based medicine. The reliance on public data for training these AI models introduces a unique set of considerations. While it enables the integration of a diverse range of information, potentially resulting in more versatile and adaptable responses, it also risks incorporating biases and inaccuracies, particularly in fields like medicine where precision is critical. This scenario highlights the necessity for rigorous evaluation and validation of content generated by ChatGPT, especially in contexts that require high accuracy and reliability. Medical professionals and researchers should engage with these AI tools cautiously, ensuring that the information aligns with established medical knowledge and practices. As research into large language models progresses, it becomes increasingly important to address these concerns. Future advancements in AI training methodologies must focus on balancing the breadth of knowledge with the accuracy needed in specialized fields. This ongoing refinement will be essential for enhancing the applicability and trustworthiness of ChatGPT and similar AI systems in professional and scientific contexts.

ChatGPT has emerged as a transformative resource within the healthcare sector, showcasing significant potential for both medical education and clinical practice. This AI-powered language model offers healthcare professionals and students an innovative way to access the latest information and developments in their fields. Its applications extend beyond simple information retrieval; it also shows promise in evaluating clinical skills and enhancing educational methodologies (28). One notable aspect of ChatGPT and similar AI chatbots is their role in improving health literacy, particularly among younger populations. These systems are increasingly being investigated for their diagnostic capabilities, with a rising interest in chatbot-based symptom checker (CSC) applications. Such tools utilize AI to mimic human interactions, providing preliminary diagnostic suggestions and fostering user-driven learning experiences (29). In clinical environments, ChatGPT has the potential to augment professional judgment and enhance patient care. Its ability to identify and flag potential warning signs or symptoms, along with its capacity to recommend appropriate medical consultations, could lead to more comprehensive healthcare delivery. However, it is essential to highlight that while ChatGPT can be a valuable supportive tool, it should not replace professional medical expertise or direct patient care. The incorporation of AI systems like ChatGPT into medical education and practice represents a significant shift in how healthcare information is accessed and utilized. These technologies can enhance traditional learning methods and support clinical decision-making. Nonetheless, their implementation raises important concerns regarding data privacy, ethical AI usage, and the necessity for ongoing validation of AI-generated information. As AI technology continues to advance, establishing robust guidelines and best practices for the integration of chatbots in medical education and clinical settings becomes increasingly vital. Future research should focus on evaluating the long-term impact of ChatGPT and similar AI tools on medical education outcomes, the accuracy of clinical decision-making, and overall patient care quality. Additionally, addressing potential biases in AI systems and ensuring equitable access to these technologies across various healthcare settings and populations are critical areas for further exploration.

ChatGPT has demonstrated important potential in medical education, especially through its performance on standardized medical examinations. Multiple studies have evaluated its capabilities across the United States Medical Licensing Exam (USMLE), revealing detailed performance metrics. ChatGPT achieved accuracy rates ranging from 41 to 65% across different USMLE steps, with some studies display specific accuracies of 55.8% on preparation questions (30, 31). The technology provides promising applications in medical education, including personalized learning experiences, enhanced clinical reasoning, and innovative educational approaches (32). Medical educators can apply ChatGPT to generate case scenarios, problem-based learning materials, and teaching content, which conserves time and increases work efficiency (33). The AI present potential in encouraging clinical decision-making, generating differential diagnoses, and facilitating simulated patient interactions (34). However, the integration of ChatGPT is not without challenges. Concerns include information accuracy, potential for artificial misperception and the risk of overreliance on technology (33). Medical institutions can carefully navigate these constraints while leveraging the technology strengths in precision medical education (32). Comparative studies have also detailed the superior performance of GPT-4, which achieved up to 86% accuracy on USMLE questions, compared to ChatGPT more moderate performance. This suggests ongoing evolution and advancement in AI-assisted medical education technologies (30).

This current review article involved a complete search of PubMed/MEDLINE, Embase, Web of Science, and Google Scholar databases. We specifically focused English-language articles and preprints published before June 2024, with a focus on ChatGPT applications in medical education. We excluded non-English studies, as well as those that focused on non-medical fields or were unrelated to medical education in practice or theory. This method ensured a precise and relevant synthesis of ChatGPT role in medical education. The review incorporated findings from peer-reviewed original, review, systematic reviews, and credible preprint articles, providing a detailed information of ChatGPT’s role in medical education.

This article aims to provide a thorough analysis of the benefits, limitations, and ethical considerations associated with the use of ChatGPT in medical education, contributing to the ongoing discussion about the responsible integration of AI in healthcare.

## 2 The ChatGPT in medical education

**Figure 2:**
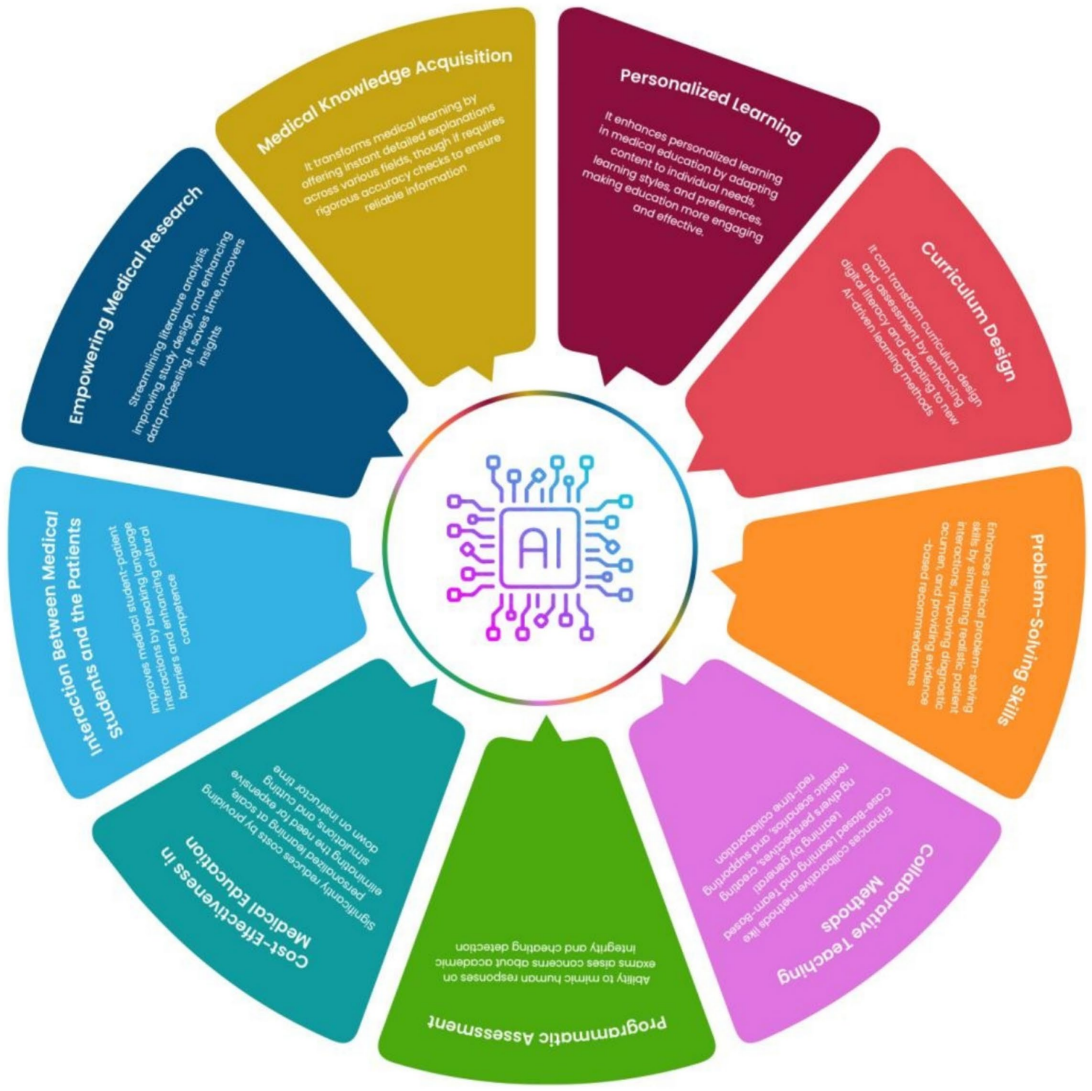
Applications of ChatGPT in medical education.

**Figure 3:**
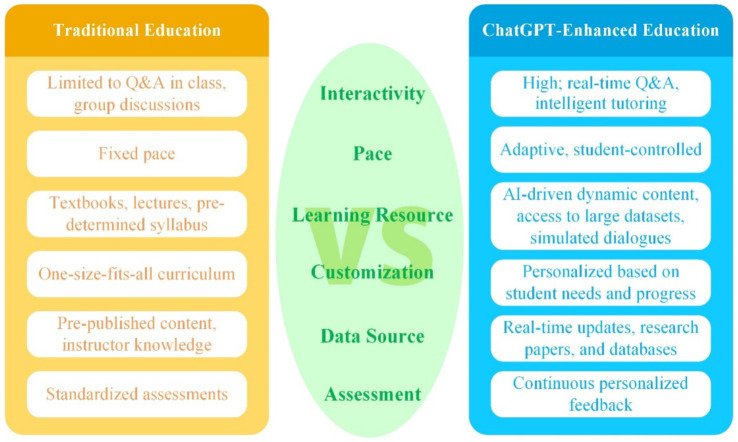
Traditional education vs. ChatGPT enhanced education medical filed.

The incorporation of artificial intelligence into medical education has announced in a new era of learning possibilities. Among the various AI tools, ChatGPT has emerged as a particularly innovative and versatile resource. This advanced language model offers a range of applications that could significantly transform medical education methodologies. One of most promising features ChatGPT is its ability to provide individualized learning experiences. By analyzing performance of each student and needs, it can offer customized educational content and provide immediate, constructive feedback. This adaptive approach enables more efficient and effective learning, accommodating the unique pace and style of each student (35). ChatGPT also shows potential in helping medical students develop crucial communication skills. Under proper academic supervision, students can use the AI to practice and improve their patient interaction techniques, learning to convey complex medical information in a clear and empathetic manner (36). Another exciting application of ChatGPT in medical education is its ability to generate realistic clinical scenarios. Recent studies have demonstrated its capacity to quickly create consistent and diverse clinical vignettes of varying complexity. This feature offers an economical method for producing valuable educational materials, potentially revolutionizing case-based learning in medical curricula (37). As AI technology continues to advance, its integration into medical education presents both opportunities and challenges. Educators and institutions must carefully consider how to effectively utilize these tools to enhance the learning experience while maintaining the integrity and quality of medical education (Figures 2, 3).

### 2.1 Revolutionizing medical knowledge acquisition

The integration of artificial intelligence into medical education has high priest in a new era of learning opportunities. ChatGPT, a sophisticated language model, has emerged as a versatile and powerful tool in this field (38). Its capacity to rapidly access and present medical information has the potential to revolutionize how students acquire and process knowledge in healthcare disciplines (39). As shown in the study by Haltaufderheide and Ranisch, ChatGPT has the capability to speedily approach and present medical content (39). Its encompassing knowledge across specialties provides an efficient learning tools, supplying succinct explanations for both basic sciences and clinical exercise. As identified by Jamal et al., the model line up with the need for accelerated information retrieval, optimizing medical education (40). However, integration of the ChatGPT necessities establishment through cross-referencing with definitive sources to avoid misinformation (41). Rapid data processing of ChatGPT enhances disease-related content generation, facilitating patients to improved communication with medical professionals (42). Experts reported that over 70% of responses on liver transplantation were highly rated, suggesting ChatGPT as a useful preliminary tool. Research in fields like periodontal disease and dermatology demonstrates ChatGPT excels at addressing patient concerns (43). Compared to routine search engines, it offers more specific, scientifically solid answers, such as in breast implant-associated lymphoma (43). It also enables patients to access medical information anytime, optimizing the geographical disparities (44). Additionally, its use in mental health allows private, subjective screenings and aids emotional awareness training (45).

ChatGPT’s extensive knowledge base, encompassing multiple medical specialties, offers a comprehensive resource for students navigating the complex landscape of medical education. From foundational sciences to clinical practice, it can provide succinct yet detailed explanations, addressing the need for efficient learning tools in contemporary education (46). The model’s ability to offer instant responses to queries about medical concepts, treatment protocols, and diagnostic criteria streamlines the learning process, aligning with the current demand for quick and effective information access in medical training (40). This immediacy of information retrieval could significantly impact how students and educators interact with medical knowledge (47). However, maintaining accuracy in medical education remains paramount. While ChatGPT excels at generating responses, it is not infallible. Its integration necessitates a rigorous validation process, involving cross-referencing with authoritative medical literature, peer-reviewed research, and established guidelines. This dual-verification approach, combining AI capabilities with human expertise, helps safeguard against misinformation (41). The role of ChatGPT as a comprehensive and immediate information resource presents an opportunity to reassess information access in medical education. Its swift retrieval and consolidation of medical knowledge, coupled with the potential for real-time updates, could significantly alter the landscape of medical learning. Nevertheless, it is crucial to acknowledge that implementing ChatGPT requires stringent quality control measures to ensure information accuracy and reliability. As educators explore its potential, they must strike a balance between technological convenience and the commitment to providing accurate, up-to-date medical information to future healthcare professionals (48). ChatGPT’s probable to generate medical misinformation poses serious risks. A study found that ChatGPT provided inaccurate information on self-managed medication abortions, incorrectly describing them as unsafe despite strong evidence supporting their safety (49). This misinformation could create unnecessary fear, stigma, and drive individuals toward riskier alternatives. Similarly, another study reported that ChatGPT failed to provide correct diagnoses in 83% of pediatric cases, highlighting its limitations in clinical decision-making and the potential for harmful delays in treatment (50). These findings underscore the threats of AI-generated misinformation in healthcare, emphasizing the need for rigorous validation and human oversight. In conclusion, while ChatGPT offers exciting possibilities for enhancing medical education, its integration must be approached with caution and critical evaluation. The focus should remain on leveraging AI as a supportive tool to augment, rather than replace, traditional learning methods and human expertise in medical education.

### 2.2 AI-driven personalized learning experiences in medical education

Personalizing learning experiences is essential in modern medical education. This section examines how ChatGPT can enhance individualized learning by adapting content to meet the unique needs, preferences, and learning styles of students. Such customization can make the educational journey more engaging and effective, signaling a shift in medical pedagogy. The diversity in students’ backgrounds, prior knowledge, and learning speeds highlights the need for tailored approaches (51). ChatGPT utilizes advanced natural language processing to analyze student inquiries and interactions, creating personalized profiles for each learner (52). These profiles inform the development of customized educational content and strategies that align with individual needs. By leveraging AI-driven personalization, ChatGPT can address varying comprehension levels within a cohort. For example, a student struggling with anatomy might receive explanations with visual aids, while a more advanced learner could engage with in-depth analyses and case studies. This tailored approach ensures that students interact with material suited to their understanding, enhancing engagement and reducing frustration. Implementing personalized learning through ChatGPT not only improves individual outcomes but also transforms medical education. It provides educators with insights into student learning patterns, enabling more effective teaching methodologies. However, it is vital to balance AI-driven personalization with human interaction, as experienced educators play a crucial role in guiding and mentoring students. The challenge is to integrate ChatGPT’s capabilities with traditional teaching methods for a holistic and effective educational experience.

In the realm of modern medical education, personalized learning has become a cornerstone of effective instruction. The ability of ChatGPT to tailor educational content to individual needs represents a significant advancement in this field. For instance, a novice student grappling with intricate anatomical structures might benefit from simplified explanations augmented by 3D models or animated sequences. In contrast, an advanced learner could be challenged with in-depth analyses of recent medical breakthroughs or complex case studies that push the boundaries of their knowledge (53). This adaptive approach ensures that each learner receives content that is neither overwhelmingly complex nor frustratingly basic, but rather perfectly aligned with their current level of understanding and learning goals (54). Such customization not only enhances comprehension but also maintains engagement and motivation throughout the learning process. Furthermore, ChatGPT’s capacity to cater to diverse learning styles marks a revolutionary step in educational technology (55). By recognizing whether a student is predominantly a visual, auditory, or kinesthetic learner, the AI can adjust its teaching methods accordingly. Visual learners might receive information through detailed infographics or color-coded diagrams. Auditory learners could benefit from spoken explanations or discussions, potentially even with varying accents or speech patterns to maintain interest. Kinesthetic learners might engage with interactive simulations or virtual labs that allow for hands-on exploration of medical concepts. This multifaceted approach to content delivery ensures that information is not just presented, but truly internalized by learners in a way that resonates with their natural learning preferences. As a result, the educational experience becomes more intuitive, enjoyable, and ultimately more effective in fostering long-term retention and practical application of medical knowledge.

The ongoing interactions of the ChatGPT with students yield valuable insights into their learning preferences, pace, and areas of interest. By analyzing patterns in students’ queries and responses over time, ChatGPT can adapt its feedback to better suit individual needs. For instance, if a student shows a consistent interest in cardiology, ChatGPT might proactively offer updates on recent cardiology research or suggest relevant resources. This tailored approach not only enhances student engagement but also fosters a sense of ownership in the learning process. Effective education relies heavily on student engagement, and ChatGPT’s dynamic, conversational nature significantly contributes to this aspect. Rather than passively consuming information, students actively participate in discussions, debates, and inquiries, transforming the learning experience into an interactive dialogue that stimulates curiosity and exploration (56). Moreover, ability of the ChatGPT to provide prompt responses to student questions creates a sense of immediacy that mirrors the real-time demands of medical practice (57). The potential of ChatGPT to enhance personalized learning in medical education represents a significant advancement in teaching strategies. By tailoring content, accommodating various learning styles, and promoting engagement through interactive conversations, ChatGPT enables a customized educational journey for each student. As medical educators strive to improve learning outcomes and equip students with versatile skills, ChatGPT emerges as a transformative tool with the potential to redefine personalized medical education (58).

The fusion of ChatGPT with immersive technologies like VR and AR presents innovative opportunities for medical education. These tools can create lifelike clinical simulations, offering students a risk-free environment to develop critical decision-making skills. ChatGPT’s ability to provide instant, personalized feedback during these simulations enhances the learning experience, creating a uniquely tailored educational journey (59). The sophisticated data analysis capabilities of ChatGPT offer deep insights into student performance. By examining learning trends over time, it can anticipate potential challenges and suggest targeted interventions. This proactive approach allows educators to address learning gaps before they become significant obstacles (60). In the rapidly advancing field of medicine, ChatGPT’s capacity for continuous learning is invaluable. By consistently incorporating the latest medical research and guidelines, it ensures students remain at the forefront of medical knowledge, preparing them for the ever-evolving healthcare landscape (61). Ultimately, the integration of ChatGPT in medical education marks a significant shift toward truly personalized learning. By harnessing AI to craft individualized educational experiences, we can cultivate a more dynamic, effective, and engaging learning environment for future healthcare professionals.

### 2.3 Using ChatGPT for curriculum design

Integrating AI technologies like ChatGPT into medical education can significantly transform curriculum design, assessment strategies, and teaching methods (Table 1). As AI becomes more prevalent in healthcare, it is crucial to equip medical students with the digital literacy and competencies necessary to use these advanced technologies. Medical educators may need to adapt their approaches to incorporate AI-based learning methods into curricula. AI tools can enhance students’ ability to acquire essential skills for future medical practice, particularly in areas where digital tools like online platforms, apps, and electronic health records are common. Students will need to develop proficiency in interpreting and applying AI-generated outputs, such as radiology reports, and communicating these findings with colleagues and patients (62). The integration of AI tools may also require a redesign of assessment strategies in medical education. Traditional methods like written exams may not adequately evaluate digital and technology literacy skills. Additionally, the potential use of AI for academic misconduct raises questions about the validity of conventional assessments such as report or essay writing. As AI transforms learning processes and clinical practice, new assessment strategies may be needed to meet the demands of the technology-driven healthcare industry. AI technologies like ChatGPT can also impact educational planning and teaching methods. Currently, AI can assist educators in drafting and brainstorming lesson plans. As AI’s teaching capabilities evolve, traditional lectures and demonstrations may become less effective. Consequently, medical educators must explore and implement teaching methods best suited for technology-enhanced learning environments (63). A systematic review led by Jörgens et al. examined 24 articles published up to February 2024, unveiling the widespread adoption of ChatGPT for curriculum design across different educational disciplines. The research demonstrated that ChatGPT display significant ability in supporting educators with various aspects of curriculum development (64). Mainly, the AI tool demonstrated expertise in generating learning activities, creating educational content, and developing assessments. The effectiveness of ChatGPT in these tasks varied considerably, with accuracy ratings ranging from 50 to 92%, contingent upon the specific nature of the task (64). By embracing AI-driven educational tools and methodologies, medical education can better prepare future healthcare professionals for the increasingly technology-dependent landscape of modern medicine.

**Table 1:** The using ChatGPT for curriculum design.

Framework	Key stages	The ChatGPT enhancement
Bloom’s taxonomy	1. Remember (recall facts and concepts)	Generates summaries, quizzes, flashcards, and mnemonics
	2. Understand (explain concepts)	Provides explanations in various difficulty levels, generates scenarios and analogies
	3. Apply (use knowledge in new situations)	Suggests case studies, real-world applications, and simulations
	4. Analyze (break down ideas into components)	Assists in developing comparative analysis questions and critical thinking exercises
	5. Evaluate (justify a decision)	Helps design debates and project reviews
	6. Create (produce original work)	Aids in brainstorming projects, simulations, and problem-based learning
Kern’s six steps	1. Problem identification	Analyses data to identify gaps in education
	2. Needs assessment	Gathers and synthesizes educational needs from literature and surveys
	3. Goals and objectives	Suggests learning objectives based on curricular needs
	4. Educational strategies	Recommends instructional methods, such as project-based learning or interactive activities
	5. Implementation	Assists in developing lesson plans, course materials, and personalized learning experiences
	6. Evaluation and feedback	Generates assessment rubrics, quizzes, and personalized feedback

### 2.4 Enhancing clinical problem-solving skills through AI-assisted simulations

The core of medical education is preparing future physicians to handle complex clinical scenarios with competence and confidence. Clinical problem-solving, a vital part of medical practice, requires strong critical thinking, diagnostic abilities, and evidence-based decision-making. Integrating artificial intelligence (AI), particularly ChatGPT, has become a powerful tool to enhance this educational process (65). ChatGPT’s AI capabilities make it a dynamic platform for simulating realistic patient interactions. By inputting hypothetical patient cases, students can engage in simulated dialogues that mimic actual clinical encounters (66). This approach moves beyond passive learning, immersing students in the complexities of patient-provider communication. ChatGPT’s adaptive responses replicate the unpredictability and variability of real clinical consultations, helping students improve their ability to think quickly, develop empathy, and understand the nuances of patient communication (67).

As students engage with ChatGPT, they develop critical communication skills essential for effective patient care, which supports a holistic approach to clinical problem-solving (56). The platform’s ability to simulate a variety of patient scenarios provides medical students with valuable opportunities to practice clinical decision-making, covering everything from routine cases to complex medical challenges (68). This diverse exposure enhances their diagnostic skills, allowing them to identify patterns, generate hypotheses, and select appropriate diagnostic tests. The iterative nature of these simulations encourages students to continually refine their diagnostic strategies, fostering resilience and a growth mindset in the face of uncertainty (69). In terms of evidence-based decision-making, ChatGPT allows students to access a vast array of medical literature, clinical guidelines, and research findings (70). When students seek information about treatment options or diagnostic criteria, ChatGPT provides evidence-based recommendations, illustrating how medical knowledge can be applied in practical scenarios. However, incorporating ChatGPT into clinical problem-solving requires careful attention to ensure the accuracy and reliability of its recommendations. This involves rigorous validation processes, human oversight, and adherence to established medical guidelines to avoid the dissemination of incorrect information (71). Additionally, it is crucial to educate students about the limitations of AI, ensuring they understand that ChatGPT should be viewed as a supportive tool rather than a replacement for their clinical judgment.

Recent research highlights the promising role of ChatGPT in clinical decision-making. A study demonstrated that ChatGPT achieved approximately 72% accuracy in overall clinical decision-making, encompassing tasks from generating possible diagnoses to final diagnoses and care management decisions (72). This performance, which is on par with that of a medical intern or resident, indicates the potential of large language models as valuable tools for clinical decision support. Furthermore, ChatGPT’s capacity to deliver unbiased responses across diverse patient demographics is particularly significant. The study found no evidence of gender bias in ChatGPT’s clinical decision-making, suggesting that such AI tools could help mitigate biases in healthcare (15). This unbiased approach could be crucial in training future physicians to provide equitable care. As medical education continues to integrate technology, ChatGPT emerges as a catalyst for enhancing clinical problem-solving skills (73–75).

A study published in May 2023 identified that ChatGPT perfectly answered seven out of fifteen practice problems and five out of fifteen transfer problems in verbal insight tasks, with its execution matching the most probable outcome for human participants across both problem sets (76). Another study found that using ChatGPT significantly improved confidence of the students in asking insightful questions, analyzing information, and understanding complex concepts, with ChatGPT usage increasing from 46 to 67% (*p* = 0.039) (77). ChatGPT with critical thinking and problem-solving frameworks to aid students solve real-time tasks, demonstrating its potential to enhance problem-solving skills in education (78). A study with 515 participants in educational settings revealed the impact of the ChatGPT on critical thinking, problem-solving, and creativity, offering insights into AI-assisted learning effect on cognitive skills development (79). By bridging the gap between theoretical knowledge and practical application, AI-assisted simulations are fostering a generation of healthcare professional’s adept at navigating complex clinical scenarios. When properly implemented and supervised, the integration of such tools has the potential to significantly improve the quality and efficiency of medical education, ultimately leading to better patient care.

### 2.5 Synergizing ChatGPT with other collaborative teaching methods

The incorporation of ChatGPT into medical education represents a substantial advancement in teaching strategies, augmenting established collaborative methods such as Case-Based Learning (CBL), Team-Based Learning (TBL), and small-group discussions. Research indicates that ChatGPT’s capacity to generate a wide range of perspectives and solutions significantly enhances the TBL experience (80). In CBL environments, ChatGPT functions as an advanced case facilitator by crafting realistic clinical scenarios, posing thought-provoking questions, and providing detailed feedback. This AI-powered approach allows for the simulation of intricate patient interactions and medical challenges, enabling students to practically apply theoretical knowledge. The model’s flexibility ensures that cases are tailored to the learners’ progressing levels of understanding, potentially encompassing a wide array of medical specialties and uncommon conditions that students might not encounter during limited clinical rotations (66, 81). Within the TBL framework, ChatGPT promotes real-time collaboration by offering immediate support and facilitating knowledge exchange among team members. It enhances group discussions by elucidating complex medical concepts and fostering critical thinking. Furthermore, ChatGPT assists in pre-class preparation by providing essential knowledge and pertinent resources for forthcoming TBL sessions. This AI-supported preparation can help level the playing field for students with diverse backgrounds and learning styles (82). Studies have shown that AI-enhanced collaborative learning can provide personalized interaction, improved group dynamics, and immediate feedback, creating a balanced ecosystem where AI augments human capabilities (83). Wiboolyasarin et al. investigated synergizing collaborative writing with AI feedback in a wiki-based environment for L2 writing improvement. The study integrated ChatGPT for written corrective feedback, revealing significant enhancements in undergraduate students’ writing proficiency (84). Pahi et al. proposed an active learning approach using TAs and generative AI for feedback in Computer Science courses. TAs assessed student progress, while ChatGPT provided examples and motivation, improving feedback quality and engagement in computing education (85).

To enhance collaborative skills, medical institutions should prioritize inter professional education, where students from various healthcare disciplines collaborate on complex cases. This method mirrors the interdisciplinary nature of current healthcare teams and equips students for real-world collaborative practice. Promoting student-led initiatives and group projects can foster leadership skills and effective communication among future healthcare professionals (86, 87). Additionally, ChatGPT can be utilized to create virtual patients or simulate healthcare scenarios, enabling students to practice decision-making in a controlled environment. This is particularly beneficial when clinical placements are limited or when students require experience with uncommon or high-risk cases. ChatGPT integration also facilitates personalized learning paths by analyzing student interactions and performance. The AI can identify knowledge gaps and suggest tailored learning resources or additional practice scenarios (88–90). This personalized approach allows students to focus on areas needing improvement while reinforcing their strengths. However, it is crucial to strike a balance, ensuring that AI complements rather than replaces essential human interactions in medical education. Educators must be trained to effectively integrate ChatGPT into their teaching methods, and students should learn to critically assess AI-generated information. Ethical considerations, such as data privacy and potential AI biases, should also be incorporated into the curriculum. This comprehensive strategy, blending AI-assisted learning with traditional collaborative methods, ensures a well-rounded educational experience. It maximizes the benefits of both individualized and team-based learning, preparing students for the complexities of modern healthcare, including the increasing role of AI in medical practice (91–93).

### 2.6 ChatGPT in programmatic assessment

Recent studies have underscored the potential implications of generative AI on high-stakes knowledge assessments in education. ChatGPT, a sophisticated language model, has shown an ability to generate responses to open-ended prompts by drawing on its extensive training on diverse online information sources. Its performance on graduate-level exams and its capability to create content that is indistinguishable from human-generated responses have raised substantial concerns among educators (94–96). In medical education, ChatGPT’s results on the United States Medical Licensing Examinations have been particularly striking, with the model achieving scores at or near the passing threshold for all three parts of the exam. Online exams have always been susceptible to cheating (97–99), and ChatGPT introduces a new dimension for accessing information. Students with access to ChatGPT during open-book exams or on unsecured computers can potentially use it to generate draft responses. For open-ended questions, ChatGPT can produce text that may be indistinguishable from that created by students, eluding conventional plagiarism detection software (100–103). This development complicates educators’ efforts to accurately assess students’ genuine understanding and work. In response to these challenges, educators are exploring various strategies to prevent students from accessing ChatGPT during exams. Existing methods may need to be updated to address the current AI landscape. While detection mechanisms for AI-generated content might become available in the future, they are not yet widely implemented. As the role of AI in education evolves, it is crucial for academic institutions to adapt their assessment practices and policies to uphold the integrity and validity of high-stakes examinations.

Educators can adopt pro-active assessment strategies to minimize the misuse of generative AI. Implementing innovative techniques such as group projects, multimedia assignments, presentations, and oral exams can be effective in various educational settings. Integrating generative AI tools into the learning and assessment processes can also enhance students’ critical evaluation skills. For instance, students can be tasked with generating responses using AI or critiquing AI-generated material, which can significantly enrich the learning experience (101). AI can play a vital role in formative assessments by analyzing data through thematic text analysis to identify misconceptions and knowledge gaps. AI-powered assessment tools can provide adaptive, personalized, and continuous feedback, similar to a tutor, thereby enhancing learning and reducing faculty workload (104). In summative assessments, AI can streamline the scoring and interpretation of performance data. For example, in written assessments with rubric-based scoring, generative AI can assist by partially automating the scoring process and suggesting narrative feedback, thus easing the burden on human raters. Although AI’s reliability in automated scoring is still developing, improvements in AI algorithms are expected to enhance the efficiency of scoring written assessments (105–107). Furthermore, AI tools can facilitate the analysis of learning trajectories, identify growth patterns, and flag students performing below competence thresholds, enabling more continuous assessment. For educators synthesizing learner performance data, AI can generate summary conclusions. For instance, generative AI could draft student assessment summaries for medical evaluations based on supervisor narratives or other data sources, aligned with specific criteria such as clerkship competencies or specialty milestones. However, these AI-generated summaries would require manual review to ensure accurate representation of the original assessment data. It is crucial to adhere to privacy requirements when using AI in educational assessments. Using open AI platforms to process individual student data protected by the Family Educational Rights and Privacy Act (FERPA) would violate current privacy regulations. Therefore, any implementation of generative AI tools must comply with local policies and legal regulations governing student data privacy and protection (108–110).

Furthermore, the cost-effectiveness of integrating ChatGPT into medical education remains ambiguous due to the absence of standardized cost calculations, including infrastructure investments, software licensing fees, and faculty training expenses (111, 112). While ChatGPT has the potential to enhance teaching methods and address academic issues more efficiently, there is limited empirical data on its long-term financial impact (111). Potential benefits such as simulating clinical environments, facilitating self-learning, and providing cost-effective learning experiences in fields like dentistry and public health have been suggested (111). However, these claims lack concrete case studies or comprehensive cost–benefit analyses. The absence of long-term projections on how ChatGPT might transform the workload of academic staff and students further complicates the assessment of its true cost-effectiveness (112). A more structured evaluation, incorporating real-world institutional data and projected long-term impacts, is necessary to determine whether ChatGPT offers a sustainable and cost-efficient model for medical education. This evaluation should include detailed cost breakdowns, long-term workload projections, and specific case calculations to illustrate potential savings and efficiency gains.

ChatGPT has emerged as a powerful tool for generating multiple-choice questions (MCQs) in medical education, offering both opportunities and challenges. Its ability to rapidly craft consistent, realistic clinical vignettes of varying complexities has garnered significant attention in the field (113, 114). Studies have shown mixed results regarding the quality of ChatGPT-generated MCQs, with some research indicating that these AI-generated questions can be comparable in quality to human-written ones, while others highlight variations in difficulty and discrimination levels4. The effectiveness of ChatGPT in generating MCQs largely depends on the prompts used, with researchers exploring various prompting strategies, including referencing specific exam styles and adopting particular personas (114). ChatGPT can be integrated into curriculum design by generating questions aligned with learning objectives, creating diverse question types beyond MCQs, and assisting in curriculum mapping to meet regulatory requirements. However, it is crucial to note that AI-generated questions should not be used directly in examinations without thorough review, and human expertise remains essential in ensuring accuracy, relevance, and quality of the questions. The technology should be seen as augmenting, not replacing, human input in question design, with the potential to significantly reduce the time educators spend on creating assessment materials (115).

### 2.7 ChatGPT and cost-effectiveness in medical education

Integrating ChatGPT into medical education offers a promising and cost-effective way to enhance teaching methods and address academic challenges. This AI-powered tool provides numerous advantages that can significantly reduce educational expenses while improving learning outcomes (116–118). One major benefit of using ChatGPT in medical education is its capacity to deliver personalized learning experiences on a large scale (119). Traditional individualized instruction methods typically require extensive human resources, making them costly and time-consuming. In contrast, ChatGPT can provide customized guidance and feedback to multiple students simultaneously, reducing the need for one-on-one tutoring sessions (120). In clinical simulations, ChatGPT shows remarkable potential. For example, in dental education, it can create realistic patient scenarios and simulate clinical environments. This allows students to practice decision-making skills without the need for expensive physical simulators or patient actors. This virtual approach not only reduces costs but also offers a safe space for students to learn from mistakes without real-world consequences (121–123).

Studies emphasize the potential of ChatGPT in medical education, highlighting its ability to enhance learning experiences and foster AI-related competencies like critical evaluation. Strengths of the ChatGPT include summarizing literature, supporting in research design, creating clinical scenarios, and facilitating virtual patient simulations, thereby enhancing problem-solving and decision-making ability (111, 124). An optimized surgical clerkship using ChatGPT significantly boosted learner satisfaction and compliance, enhancing educational outcomes (125). Likewise, a randomized trial in orthopedic education reported higher student engagement in pre- and post-class activities, with higher satisfaction in course organization (126). In Saudi Arabia, students appreciated ChatGPT in remote learning, noting its role in personalized learning, communication skill enhancement, and interactive assessments (127).

ChatGPT’s role in medical education extends well beyond clinical simulations. In public health, it aids in teaching complex statistical analysis by offering step-by-step guidance, thus reducing the need for costly software and extensive instructor time (128). Its ability to review and provide feedback on assignments enhances learning and reduces instructor workload by offering detailed critiques and additional resources (69). ChatGPT’s 24/7 accessibility promotes self-directed learning and reduces scheduled classroom time, leading to more efficient use of resources and potentially lower program costs (129). In research education, it aids in literature reviews, hypothesis generation, and experimental design, streamlining these processes and making research education more cost-effective (130). Despite these benefits, careful implementation is crucial. Institutions must consider initial AI infrastructure costs, ongoing maintenance, and faculty training. ChatGPT should complement, not replace, traditional teaching methods, especially where human empathy and real-world experience are vital (69, 111, 124–132).

### 2.8 Enhancing the quality of interaction between medical students and the patients

Incorporating advanced language models like ChatGPT into medical education represents a significant advancement in improving interactions between students and patients. This technology addresses the longstanding issue of language barriers in clinical settings with its high accuracy and multilingual capabilities (133, 134). As medical students increasingly encounter patients from diverse cultural and linguistic backgrounds, effective communication becomes crucial. ChatGPT offers real-time, precise translations in multiple languages, facilitating better communication and enhancing both the accuracy of medical histories and patient experiences (135–137). Beyond translation, ChatGPT also contributes to cultural competence. It assists students in understanding cultural contexts and communication styles, helping build rapport and trust with patients from varied backgrounds, which is essential for patient-centered care (138, 139). The constant availability of ChatGPT reduces delays and misunderstandings in care. Integrating this tool into medical curricula also eases the educational load on clinical departments. Rather than investing heavily in language training or interpretation services, educators can concentrate on core medical skills, knowing that students have reliable language support (140). Furthermore, ChatGPT serves as a valuable learning resource, aiding students in developing language skills and cultural awareness over time. Interacting with the tool exposes students to medical terminology in various languages and different cultural approaches to healthcare, enhancing their overall competence as future healthcare professionals (141, 142) The use of ChatGPT aligns with the increasing role of technology in healthcare, preparing students for a future where digital tools are integral to patient care and developing technological literacy that will benefit them in their careers.

### 2.9 Empowering medical research with ChatGPT

ChatGPT is revolutionizing medical research by offering innovative ways to streamline processes and speed up discoveries. This AI tool is transforming how researchers approach their work across various aspects of the field. The integration of ChatGPT in medical research complements the standards set by organizations like WFME and CanMEDS. These bodies advocate for a holistic approach to medical education, and ChatGPT supports this by bridging the gap between research and practical training (143). A key benefit of ChatGPT is its ability to efficiently analyze vast amounts of scientific literature. This addresses the time-consuming and error-prone nature of manual literature reviews (144). ChatGPT’s language processing capabilities allow researchers to quickly identify relevant studies, extract crucial information, and synthesize findings. This not only saves time but may also uncover insights that might be overlooked through traditional methods (17).

ChatGPT’s natural language processing abilities significantly enhance medical research by quickly reviewing and synthesizing information from numerous sources. This capability provides researchers with comprehensive summaries and highlights key findings, saving time and ensuring access to a broader range of relevant information, which leads to more informed hypotheses and study designs (145). In terms of study design, ChatGPT aids researchers by generating research proposals based on existing literature and current trends. It suggests novel approaches or refinements to experimental designs, which is particularly beneficial for early-career researchers or those exploring new areas (146, 147). ChatGPT excels in data analysis by processing large datasets, identifying patterns, and recommending appropriate statistical methods. Its ability to manage complex data structures and uncover potential correlations or anomalies enhances the quality of research findings (148, 149). Additionally, ChatGPT assists in data visualization by proposing effective ways to present results graphically, which is crucial for communicating findings to peers and the public (150, 151). Furthermore, ChatGPT supports interdisciplinary approaches in medical research. Its extensive knowledge base helps make connections across different fields, potentially uncovering insights that specialists might miss (41, 152). ChatGPT’s multilingual capabilities also facilitate global collaboration by breaking down language barriers, enabling researchers worldwide to share and access information more easily, thus fostering international medical advancement.

## 3 Challenges and limitations of ChatGPT in medical mentorship

**Figure 4:**
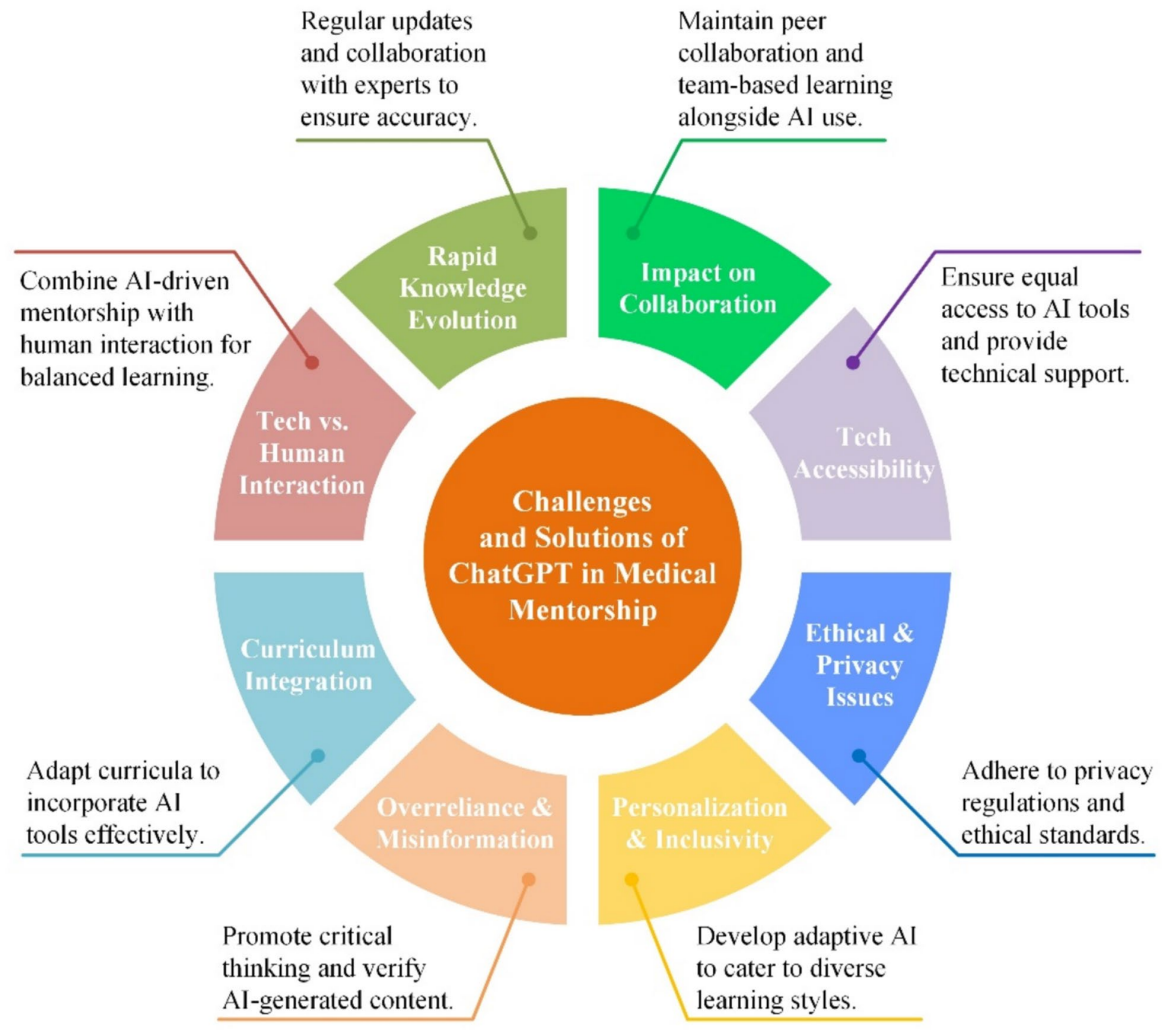
Challenges and solutions of ChatGPT in medical mentorship.

The integration of ChatGPT into medical education presents a paradigm shift in learning methodologies. While its potential benefits are substantial, the challenges it poses are equally significant and multifaceted (Figure 4). This analysis delves into the complexities of implementing ChatGPT in medical education, exploring both the obstacles and the opportunities they present.

### 3.1 Rapid evolution of medical knowledge

The rapid evolution of the medical field poses a significant challenge for integrating ChatGPT into medical education. Continuous advancements in research, treatments, and guidelines require frequent updates to ensure that students receive accurate and current information. Updating ChatGPT’s knowledge base is a time-consuming process that demands careful verification to maintain the reliability of the information provided. The fast pace of medical developments can lead to outdated information, highlighting the need for students to critically assess AI-generated content. To overcome these challenges, collaboration among medical professionals, AI developers, and educational institutions is essential. Establishing efficient update processes and verification protocols will help ensure that ChatGPT remains a valuable resource in medical education while keeping pace with ongoing changes in the healthcare landscape (153, 154).

### 3.2 Balancing technology and human interaction

While ChatGPT offers personalized guidance and enhances problem-based learning (PBL), it cannot replace the crucial aspects of human interaction in medical education (155). Educators must strike a delicate balance between leveraging AI technology and preserving essential human-to-human interactions (156–158). The irreplaceable value of patient-based learning, teamwork, and practical clinical experiences must be emphasized alongside the integration of AI tools like ChatGPT.

### 3.3 Curriculum integration and pedagogical challenges

Incorporating ChatGPT into existing medical curricula presents several challenges that require careful consideration and planning. Educators face the task of seamlessly embedding AI-driven components into their teaching methods while ensuring alignment with overall learning objectives (159, 160). This integration demands significant time investment and may necessitate redesigning certain aspects of the curriculum to effectively utilize ChatGPT’s capabilities.

### 3.4 Risks of overreliance and misinformation

The convenience of ChatGPT comes with potential drawbacks that could impact the quality of medical education. Over reliance on AI-generated responses may hinder the development of critical thinking skills among students. Additionally, the potential for ChatGPT to provide inconsistent or occasionally inaccurate information poses a risk to student learning. Establishing robust mechanisms for error detection and correction is crucial to mitigate these risks (161, 162).

### 3.5 Personalization and inclusivity

Tailoring ChatGPT to individual student needs presents both a challenge and an opportunity in medical education. Developing sophisticated algorithms that can adapt to each student’s learning pace and style is essential for effective personalized education (163, 164). Moreover, ensuring that ChatGPT can accommodate diverse cultural backgrounds and learning preferences is crucial for creating an inclusive learning environment.

### 3.6 Ethical and privacy considerations

The use of AI in medical education raises significant ethical concerns that must be carefully addressed (165, 166). Handling patient data in an educational context while safeguarding privacy is a complex issue that requires strict adherence to regulatory and ethical guidelines. To reduce the risks of inaccuracy and overreliance on AI in medical education, various fundamental recommendations should be applied. First, medical curricula must focus on critical thinking skills to ensure that students do not become excessively dependent on AI tools, which can lead to a reduce in necessary clinical intelligent capabilities (167). This can be accomplishing by incorporating training that promote students to critically evaluate AI-generated knowledge. Moreover, curriculum improvement should include detailed education on AI technologies, addressing their constraints, biases, and ethical outcomes. Transparency is also important; students should be taught to confirm AI results against authoritative sources to mitigate the risk of inaccuracy (168). Lastly, ongoing cooperation among educators, healthcare professionals, and policymakers is necessary for set up road map and best practices for AI integration in medical education. By emphasizing critical assessment and ethical principles, the medical education system can more effectively prepare future healthcare professionals to manage the intricacies of AI in their practice (169).

### 3.7 Technological accessibility and infrastructure

The effective implementation of ChatGPT in medical education depends on technological infrastructure and accessibility. Ensuring equal access to AI tools across different regions and socioeconomic backgrounds is crucial for maintaining equity in medical education. Institutions must also provide adequate technical support and resources to facilitate the smooth integration of ChatGPT into their educational programs (170, 171).

### 3.8 Impact on traditional collaborative learning methods

Incorporating ChatGPT into medical education could impact the effectiveness of collaborative learning methods, which are vital to training future healthcare professionals. Although ChatGPT can enhance approaches like problem-based learning (PBL), case-based learning (CBL), and team-based learning (TBL), it is crucial to ensure that peer interaction and collaborative problem-solving are not compromised (172, 173). Effective strategies must be developed to integrate AI assistance while maintaining the benefits of team dynamics.

While ChatGPT has significant potential to transform medical education, its implementation comes with several challenges. Addressing these challenges requires a comprehensive approach involving collaboration among educators, technologists, ethicists, and policymakers. By carefully addressing these issues, the medical education community can effectively harness AI’s capabilities to enhance learning outcomes, while preserving the critical human aspects of medical training.

## 4 Future directions of ChatGPT in medical mentorship

The adoption of ChatGPT in medical education offers exciting possibilities but also brings significant challenges that demand careful consideration. In the rural and resource-constrained medical mentorship, ChatGPT provides significant potential for innovation. This AI technology can transform admittance to learning by providing personalized experiences tailored to underserved graduates (174, 175). By generating case scenarios, quizzes, and learning materials, ChatGPT intensify self-directed learning and enhances educational quality, handling the shortage of specialized instructors (176). Furthermore, it helps rural healthcare professionals in clinical decision-making by providing evidence-based facts and crafting differential diagnoses, particularly where specialists are scarce (177). ChatGPT also assist ongoing professional development by organizing current research (175). Additionally, it can help handle mental health challenges in resource-limited domain by supplying anonymous assistance and connecting individuals to local services. However, careful integration is necessary and developments should focus on improving content quality, minimizing depends on technology, and guaranteeing users receive training in critical evaluation to maximize effectiveness in rural healthcare settings (178).

As we move forward, it is essential to tackle existing limitations while exploring new ways to enhance the learning experience for medical students. To address potential issues, implementing proper guidelines and oversight is crucial. Students need thorough training not just in using AI tools, but also in developing strong interpersonal skills for effective patient care. Ensuring equal access to these technologies across diverse student groups is vital for inclusive medical education. Future studies should investigate how ChatGPT impacts various learning approaches, including Problem-Based, Team-Based, and Case-Based Learning. Developing strategies to balance AI assistance with collaborative skills is necessary to prepare students for real-world healthcare scenarios. There is an urgent need to improve ChatGPT’s ability to provide current, contextually relevant medical information. This involves creating systems for timely knowledge updates and refining data curation processes. Addressing ethical concerns and privacy issues is also critical, requiring robust protocols to protect patient information while integrating AI into educational platforms. Exploring personalized learning approaches with ChatGPT offers promising opportunities. Research into adaptive algorithms and innovative teaching methods tailored to individual learning styles could transform medical education, potentially revolutionizing how future healthcare professionals acquire and retain knowledge.

Furthermore, there is a need for research on improving ChatGPT’s error identification and correction mechanisms, ensuring the highest level of accuracy and reliability in medical content. This is particularly important given the critical nature of healthcare information and the potential consequences of misinformation. Another crucial area for future development is enhancing ChatGPT’s cultural sensitivity and inclusivity in medical education. By acknowledging the diversity of student backgrounds and learning requirements, we can ensure that AI-assisted education is not only effective but also equitable and respectful of cultural differences. This holistic approach ensures that ChatGPT not only provides accurate medical information but also aligns with the broader goals of medical education in promoting collaboration, ethical considerations, and cultural competence. As we continue to integrate ChatGPT and similar AI technologies into medical education, it will be essential to continuously evaluate their impact on learning outcomes, clinical skills development, and overall educational effectiveness. This ongoing assessment will help identify areas for improvement and guide the evolution of AI-assisted medical education. By addressing these multifaceted challenges and opportunities, we can work toward a future where ChatGPT and other AI tools seamlessly complement traditional teaching methods, enhancing the quality and accessibility of medical education while preparing students for the technologically advanced healthcare landscape they will enter upon graduation.

## 5 Conclusion

ChatGPT offers significant benefits to medical education, enhancing clinical reasoning skills, enabling personalized learning experiences, and supporting medical research. Its ability to process vast amounts of information can help deliver more precise, up-to-date medical education. However, integrating this AI tool effectively requires a careful, balanced approach that prioritizes ethical considerations and maintains a human-centered focus in healthcare education. To achieve this balance, medical educators should implement strategies that complement AI capabilities with traditional teaching methods. This includes customizing learning pathways to individual student needs while also incorporating group activities to foster collaboration. Assigning team projects that utilize ChatGPT can help students learn to work effectively with AI tools in a collaborative setting. It is crucial to provide clear guidance on appropriate ChatGPT usage and to emphasize the importance of ethical decision-making and critical thinking in medical practice. By adopting these approaches, medical schools can create a comprehensive learning environment that leverages ChatGPT’s strengths while nurturing essential human skills. This balanced integration prepares future healthcare professionals to think independently, work collaboratively, and use AI tools responsibly. Ultimately, this approach optimizes ChatGPT’s role in medical education while preserving the field’s integrity and ensuring that graduates are well-equipped to provide high-quality, empathetic patient care in an increasingly technology-driven healthcare landscape.
